# Supplementary material for: Depressive-like Behavior Is Accompanied by Prefrontal Cortical Innate Immune Fatigue and Dendritic Spine Losses after HIV-1 Tat and Morphine Exposure
Source: Viruses. 2023 Feb 21;15(3):590. doi: 10.3390/v15030590 (PMC10052300; doi:10.3390/v15030590)
Supplement: Supplementary file 1 [file viruses-15-00590-s001.zip › viruses-2159149-supplementary.pdf]

Depressive-Like Behavior Is Accompanied by Prefrontal Cortical Innate Immune Fatigue and Dendritic Spine Losses after HIV-1 Tat and Morphine Exposure

Sara R. Nass [1], Yun K. Hahn [2], Michael Ohene-Nyako [1], Virginia D. McLane [1], M. Imad Damaj [1], Leroy R. Thacker II [3], Pamela E. Knapp [1,2,4] and Kurt F. Hauser [1,2,4,*]

Table S1. Correlations between chemokines and cytokines and behavioral outcomes not graphically displayed in Fig. 6.

Variables	Correlation	Degrees of Freedom	Z-Value	P-Value	FDR P-Value
% burrowed, CCL11	-0.159	11	-0.507	0.6123	0.9546
% burrowed, G-CSF	-0.15	11	-0.477	0.6331	0.9546
% burrowed, GM-CSF	-0.098	11	-0.31	0.7565	0.9546
% burrowed, IFN-g	-0.044	11	-0.139	0.8895	0.9546
% burrowed, IL-1a	-0.112	11	-0.356	0.7219	0.9546
% burrowed, IL-1b	-0.039	11	-0.125	0.9007	0.9546
% burrowed, IL-2	-0.053	11	-0.169	0.8662	0.9546
% burrowed, IL-3	-0.242	11	-0.78	0.4353	0.9546
% burrowed, IL-4	0.297	11	0.967	0.3335	0.9546
% burrowed, IL-5	0.059	11	0.187	0.8514	0.9546
% burrowed, IL-9	0.343	11	1.13	0.2583	0.9546
% burrowed, IL-10	-0.43	11	-1.455	0.1457	0.9546
% burrowed, IL-12(p40)	-0.004	11	-0.012	0.9908	0.9908
% burrowed, IL-12(p-70)	-0.071	11	-0.224	0.8227	0.9546
% burrowed, IL-13	-0.067	11	-0.214	0.8308	0.9546
% burrowed, IL-17A	-0.083	11	-0.262	0.7934	0.9546
% burrowed, CXCL1	0.213	11	0.686	0.493	0.9546
% burrowed, CCL2	-0.035	11	-0.109	0.9131	0.9546
% burrowed, CCL3	0.262	11	0.847	0.3969	0.9546
% burrowed, CCL4	-0.129	11	-0.409	0.6822	0.9546
% burrowed, CCL5	-0.416	11	-1.401	0.1613	0.9546
% burrowed, TNFa	-0.138	11	-0.44	0.6603	0.9546
% Nestlet Shredded, CCL11	-0.186	11	-0.594	0.5527	0.9787
% Nestlet Shredded, G-CSF	0.085	11	0.271	0.7865	0.9787
% Nestlet Shredded, GM-CSF	0.204	11	0.654	0.5129	0.9787
% Nestlet Shredded, IFN-g	0.421	11	1.419	0.156	0.9787
% Nestlet Shredded, IL-1a	-0.127	11	-0.405	0.6854	0.9787
% Nestlet Shredded, IL-1b	0.04	11	0.127	0.8991	0.9787
% Nestlet Shredded, IL-2	0.063	11	0.198	0.8427	0.9787
% Nestlet Shredded, IL-3	0.025	11	0.079	0.9369	0.9787
% Nestlet Shredded, IL-4	0.142	11	0.451	0.652	0.9787
% Nestlet Shredded, IL-5	0.283	11	0.92	0.3573	0.9787

% Nestlet Shredded, IL-6	0.333	11	1.096	0.2731	0.9787
% Nestlet Shredded, IL-9	0.326	11	1.07	0.2848	0.9787
% Nestlet Shredded, IL-10	-0.071	11	-0.225	0.8222	0.9787
% Nestlet Shredded, IL-12(p40)	-0.095	11	-0.301	0.7633	0.9787
% Nestlet Shredded, IL-12(p-70)	0.193	11	0.62	0.5355	0.9787
% Nestlet Shredded, IL-13	-0.088	11	-0.279	0.7799	0.9787
% Nestlet Shredded, IL-17A	0.217	11	0.697	0.4857	0.9787
% Nestlet Shredded, CXCL1	0.154	11	0.492	0.6229	0.9787
% Nestlet Shredded, CCL2	0.008	11	0.027	0.9787	0.9787
% Nestlet Shredded, CCL3	0.1	11	0.317	0.7513	0.9787
% Nestlet Shredded, CCL4	0.276	11	0.894	0.3711	0.9787
% Nestlet Shredded, CCL5	-0.011	11	-0.034	0.9733	0.9787
% Nestlet Shredded, TNFa	0.368	11	1.223	0.2215	0.9787
Sucrose Time (s), CCL11	-0.25	11	-0.807	0.4198	0.6175
Sucrose Time (s), G-CSF	-0.266	11	-0.86	0.3896	0.6175
Sucrose Time (s), GM-CSF	-0.243	11	-0.785	0.4326	0.6175
Sucrose Time (s), IFN-g	-2.20E-04	11	-0.001	0.9994	0.9994
Sucrose Time (s), IL-1a	-0.271	11	-0.878	0.3797	0.6175
Sucrose Time (s), IL-1b	-0.345	11	-1.137	0.2557	0.6175
Sucrose Time (s), IL-2	-0.257	11	-0.83	0.4066	0.6175
Sucrose Time (s), IL-3	-0.468	11	-1.607	0.1082	0.6175
Sucrose Time (s), IL-4	-0.432	11	-1.461	0.144	0.6175
Sucrose Time (s), IL-5	-0.265	11	-0.859	0.3904	0.6175
Sucrose Time (s), IL-6	-0.165	11	-0.525	0.5995	0.7660
Sucrose Time (s), IL-9	-0.128	11	-0.405	0.6852	0.8294
Sucrose Time (s), IL-12(p40)	0.011	11	0.036	0.9712	0.9994
Sucrose Time (s), IL-12(p-70)	0.101	11	0.321	0.7481	0.8604
Sucrose Time (s), IL-13	-0.364	11	-1.208	0.227	0.6175
Sucrose Time (s), IL-17A	-0.231	11	-0.745	0.4564	0.6175
Sucrose Time (s), CXCL1	-0.302	11	-0.987	0.3237	0.6175
Sucrose Time (s), CCL2	-0.264	11	-0.854	0.3929	0.6175
Sucrose Time (s), CCL3	-0.046	11	-0.147	0.8834	0.9676
Sucrose Time (s), CCL4	-0.436	11	-1.479	0.1393	0.6175
Sucrose Time (s), CCL5	-0.477	11	-1.642	0.1006	0.6175
Sucrose Time (s), TNFa	0.41	11	1.377	0.1685	0.6175
Sucrose latency (s), CCL11	0.164	11	0.523	0.6007	0.7272
Sucrose latency (s), G-CSF	0.317	11	1.038	0.2993	0.4985
Sucrose latency (s), GM-CSF	0.468	11	1.604	0.1088	0.4965
Sucrose latency (s), IFN-g	0.069	11	0.218	0.8275	0.8651
Sucrose latency (s), IL-1a	0.28	11	0.908	0.3638	0.4985
Sucrose latency (s), IL-1b	0.322	11	1.056	0.2908	0.4985
Sucrose latency (s), IL-2	0.243	11	0.783	0.4337	0.5542
Sucrose latency (s), IL-3	0.406	11	1.364	0.1727	0.4965
Sucrose latency (s), IL-4	0.531	11	1.869	0.0616	0.4726
Sucrose latency (s), IL-5	0.417	11	1.406	0.1597	0.4965
Sucrose latency (s), IL-6	0.13	11	0.414	0.6788	0.7806
Sucrose latency (s), IL-9	0.307	11	1.002	0.3162	0.4985
Sucrose latency (s), IL-10	0.408	11	1.371	0.1703	0.4965

Sucrose latency (s), IL-12(p40)	-0.096	11	-0.305	0.7601	0.8325
Sucrose latency (s), IL-12(p-70)	-0.277	11	-0.899	0.3685	0.4985
Sucrose latency (s), IL-13	0.434	11	1.469	0.1419	0.4965
Sucrose latency (s), IL-17A	0.369	11	1.224	0.2208	0.4985
Sucrose latency (s), CCL2	0.28	11	0.909	0.3634	0.4985
Sucrose latency (s), CCL3	0.301	11	0.983	0.3258	0.4985
Sucrose latency (s), CCL4	0.541	11	1.914	0.0556	0.4726
Sucrose latency (s), CCL5	0.363	11	1.203	0.229	0.4985
Sucrose latency (s), TNFa	-0.05	11	-0.159	0.874	0.8740
NISH Mobility (s), CCL11	-0.07	11	-0.223	0.8234	0.9985
NISH Mobility (s), G-CSF	0.203	11	0.649	0.516	0.9985
NISH Mobility (s), GM-CSF	0.137	11	0.438	0.6617	0.9985
NISH Mobility (s), IFN-g	-0.071	11	-0.225	0.8219	0.9985
NISH Mobility (s), IL-1a	-0.347	11	-1.146	0.2519	0.9985
NISH Mobility (s), IL-1b	-0.105	11	-0.332	0.7399	0.9985
NISH Mobility (s), IL-2	-0.067	11	-0.211	0.8331	0.9985
NISH Mobility (s), IL-3	0.3	11	0.979	0.3275	0.9985
NISH Mobility (s), IL-4	-0.124	11	-0.394	0.6939	0.9985
NISH Mobility (s), IL-5	0.001	11	0.002	0.9985	0.9985
NISH Mobility (s), IL-6	0.033	11	0.106	0.9158	0.9985
NISH Mobility (s), IL-9	-0.429	11	-1.449	0.1474	0.9985
NISH Mobility (s), IL-10	0.484	11	1.672	0.0946	0.9985
NISH Mobility (s), IL-12(p40)	0.015	11	0.048	0.9617	0.9985
NISH Mobility (s), IL-12(p-70)	-0.04	11	-0.128	0.8983	0.9985
NISH Mobility (s), IL-13	-0.027	11	-0.084	0.9328	0.9985
NISH Mobility (s), IL-17A	0.181	11	0.578	0.5636	0.9985
NISH Mobility (s), CXCL1	-0.009	11	-0.029	0.9766	0.9985
NISH Mobility (s), CCL2	0.336	11	1.104	0.2697	0.9985
NISH Mobility (s), CCL3	-0.037	11	-0.118	0.9065	0.9985
NISH Mobility (s), CCL4	0.095	11	0.302	0.7628	0.9985
NISH Mobility (s), CCL5	0.029	11	0.091	0.9273	0.9985
NISH Mobility (s), TNFa	-0.066	11	-0.207	0.8356	0.9985
NISH Rearing (s), CCL11	0.017	11	0.055	0.9562	0.9775
NISH Rearing (s), G-CSF	0.056	11	0.177	0.8593	0.9775
NISH Rearing (s), GM-CSF	-0.513	11	-1.793	0.073	0.7084
NISH Rearing (s), IFN-g	-0.137	11	-0.436	0.6632	0.8972
NISH Rearing (s), IL-1a	0.222	11	0.713	0.476	0.7820
NISH Rearing (s), IL-1b	0.466	11	1.596	0.1106	0.7084
NISH Rearing (s), IL-2	0.451	11	1.537	0.1242	0.7084
NISH Rearing (s), IL-3	-0.295	11	-0.96	0.337	0.7084
NISH Rearing (s), IL-4	0.259	11	0.837	0.4027	0.7453
NISH Rearing (s), IL-5	0.021	11	0.066	0.947	0.9775
NISH Rearing (s), IL-9	0.009	11	0.028	0.9775	0.9775
NISH Rearing (s), IL-10	-0.363	11	-1.204	0.2288	0.7084
NISH Rearing (s), IL-12(p40)	-0.294	11	-0.956	0.3388	0.7084
NISH Rearing (s), IL-12(p-70)	-0.249	11	-0.804	0.4213	0.7453
NISH Rearing (s), IL-13	0.308	11	1.006	0.3143	0.7084
NISH Rearing (s), IL-17A	-0.337	11	-1.109	0.2676	0.7084

NISH Rearing (s), CXCL1	0.062	11	0.196	0.8443	0.9775
NISH Rearing (s), CCL2	-0.372	11	-1.236	0.2164	0.7084
NISH Rearing (s), CCL3	-0.158	11	-0.504	0.6142	0.8830
NISH Rearing (s), CCL4	-0.185	11	-0.593	0.5534	0.8486
NISH Rearing (s), CCL5	0.046	11	0.145	0.8849	0.9775
NISH Rearing (s), TNFa	-0.346	11	-1.14	0.2542	0.7084
Mobility FST, CCL11	0.563	9	1.802	0.0716	0.9808
Mobility FST, G-CSF	-0.127	9	-0.361	0.7185	0.9808
Mobility FST, GM-CSF	-0.2	9	-0.573	0.5665	0.9808
Mobility FST, IFN-g	0.009	9	0.024	0.9808	0.9808
Mobility FST, IL-1a	0.139	9	0.395	0.6929	0.9808
Mobility FST, IL-1b	0.341	9	1.004	0.3154	0.9808
Mobility FST, IL-2	0.341	9	1.004	0.3154	0.9808
Mobility FST, IL-3	-0.207	9	-0.593	0.5531	0.9808
Mobility FST, IL-4	-0.04	9	-0.114	0.9095	0.9808
Mobility FST, IL-5	-0.08	9	-0.226	0.821	0.9808
Mobility FST, IL-6	0.39	9	1.163	0.2447	0.9808
Mobility FST, IL-9	0.459	9	1.403	0.1606	0.9808
Mobility FST, IL-10	0.086	9	0.245	0.8065	0.9808
Mobility FST, IL-12(p40)	0.122	9	0.348	0.7282	0.9808
Mobility FST, IL-12(p-70)	0.405	9	1.215	0.2244	0.9808
Mobility FST, IL-13	0.255	9	0.738	0.4603	0.9808
Mobility FST, IL-17A	-0.278	9	-0.808	0.4192	0.9808
Mobility FST, CXCL1	-0.027	9	-0.076	0.9395	0.9808
Mobility FST, CCL2	0.138	9	0.392	0.6948	0.9808
Mobility FST, CCL3	0.061	9	0.173	0.8624	0.9808
Mobility FST, CCL4	0.248	9	0.717	0.4735	0.9808
Mobility FST, CCL5	0.391	9	1.167	0.2431	0.9808
Mobility FST, TNFa	0.313	9	0.915	0.3602	0.9808
Food time (s), CCL11	0.352	9	1.041	0.298	0.6232
Food time (s), G-CSF	0.258	9	0.747	0.4551	0.6608
Food time (s), GM-CSF	0.223	9	0.643	0.5205	0.7042
Food time (s), IFN-g	-0.035	9	-0.1	0.9206	0.9206
Food time (s), IL-1a	0.412	9	1.239	0.2154	0.6126
Food time (s), IL-1b	0.302	9	0.882	0.3775	0.6608
Food time (s), L-2	0.302	9	0.882	0.3775	0.6608
Food time (s), IL-3	0.446	9	1.356	0.175	0.6126
Food time (s), IL-4	0.083	9	0.236	0.8137	0.8912
Food time (s), IL-5	0.505	9	1.574	0.1155	0.6126
Food time (s), IL-6	0.066	9	0.186	0.8526	0.8914
Food time (s), IL-9	-0.094	9	-0.267	0.7896	0.8912
Food time (s), IL-10	0.545	9	1.727	0.0841	0.6126
Food time (s), IL-12(p40)	0.393	9	1.176	0.2397	0.6126
Food time (s), L-12(p-70)	-0.152	9	-0.434	0.6642	0.8487
Food time (s), IL-13	0.101	9	0.287	0.7745	0.8912
Food time (s), IL-17A	0.256	9	0.739	0.4597	0.6608
Food time (s), CXCL1	0.429	9	1.298	0.1944	0.6126
Food time (s), CCL2	0.517	9	1.619	0.1055	0.6126

Food time (s), CCL3	0.368	9	1.092	0.2748	0.6232
Food time (s), CCL4	0.447	9	1.359	0.1742	0.6126
Food time (s), CCL5	0.276	9	0.802	0.4226	0.6608
Food time (s), TNFa	-0.559	9	-1.787	0.0739	0.6126
Food latency (s), CCL11	-0.403	9	-1.208	0.2272	0.9933
Food latency (s), G-CSF	-0.201	9	-0.575	0.5651	0.9933
Food latency (s), GM-CSF	-0.06	9	-0.169	0.866	0.9933
Food latency (s), IFN-g	-0.012	9	-0.033	0.9735	0.9933
Food latency (s), IL-1a	-0.129	9	-0.366	0.7145	0.9933
Food latency (s), IL-1b	-0.451	9	-1.374	0.1693	0.9933
Food latency (s), IL-2	-0.451	9	-1.374	0.1693	0.9933
Food latency (s), IL-3	0.003	9	0.008	0.9933	0.9933
Food latency (s), IL-4	0.082	9	0.233	0.8158	0.9933
Food latency (s), IL-5	-0.291	9	-0.846	0.3974	0.9933
Food latency (s), IL-6	0.202	9	0.578	0.563	0.9933
Food latency (s), IL-9	0.03	9	0.086	0.9317	0.9933
Food latency (s), IL-10	-0.152	9	-0.435	0.6639	0.9933
Food latency (s), IL-12(p40)	0.218	9	0.628	0.5301	0.9933
Food latency (s), IL-12(p-70)	-0.012	9	-0.033	0.9739	0.9933
Food latency (s), IL-13	-0.231	9	-0.665	0.5063	0.9933
Food latency (s), IL-17A	-0.139	9	-0.397	0.6913	0.9933
Food latency (s), CXCL1	-0.118	9	-0.335	0.7377	0.9933
Food latency (s), CCL2	-0.178	9	-0.509	0.6108	0.9933
Food latency (s), CCL3	-0.011	9	-0.031	0.9751	0.9933
Food latency (s), CCL4	-0.322	9	-0.945	0.3447	0.9933
Food latency (s), CCL5	-0.371	9	-1.102	0.2706	0.9933
Food latency (s), TNFa	0.532	9	1.675	0.0939	0.9933
NSF Mobility (s), G-CSF	0.05	9	0.14	0.8885	0.9192
NSF Mobility (s), GM-CSF	-0.242	9	-0.697	0.4855	0.7711
NSF Mobility (s), IFN-g	-0.221	9	-0.635	0.5254	0.7711
NSF Mobility (s), IL-1a	-0.192	9	-0.55	0.5823	0.7711
NSF Mobility (s), IL-1b	-0.133	9	-0.38	0.7041	0.7711
NSF Mobility (s), IL-2	-0.133	9	-0.38	0.7041	0.7711
NSF Mobility (s), IL-3	-0.172	9	-0.491	0.6233	0.7711
NSF Mobility (s), IL-4	0.223	9	0.642	0.5209	0.7711
NSF Mobility (s), IL-5	-0.519	9	-1.626	0.1039	0.7711
NSF Mobility (s), IL-6	-0.141	9	-0.401	0.6884	0.7711
NSF Mobility (s), IL-9	-0.201	9	-0.576	0.5646	0.7711
NSF Mobility (s), IL-10	-0.275	9	-0.799	0.4243	0.7711
NSF Mobility (s), L-12(p40)	-0.346	9	-1.022	0.3066	0.7711
NSF Mobility (s), IL-12(p-70)	-0.22	9	-0.632	0.5273	0.7711
NSF Mobility (s), IL-13	0.036	9	0.101	0.9192	0.9192
NSF Mobility (s), IL-17A	-0.205	9	-0.589	0.5556	0.7711
NSF Mobility (s), CXCL1	-0.277	9	-0.804	0.4213	0.7711
NSF Mobility (s), CCL2	-0.471	9	-1.446	0.1482	0.7711
NSF Mobility (s), CCL3	-0.399	9	-1.196	0.2317	0.7711
NSF Mobility (s), CCL4	-0.203	9	-0.583	0.56	0.7711
NSF Mobility (s), CCL5	-0.226	9	-0.651	0.5151	0.7711

NSF Mobility (s), TNFa	0.344	9	1.015	0.3099	0.7711
NSF Rearing (s), G-CSF	0.276	9	0.802	0.4224	0.9574
NSF Rearing (s), GM-CSF	0.014	9	0.039	0.9691	0.9784
NSF Rearing (s), FN-g	-0.128	9	-0.363	0.7163	0.9784
NSF Rearing (s), IL-1a	-0.233	9	-0.672	0.5017	0.9574
NSF Rearing (s), IL-1b	-0.171	9	-0.49	0.6244	0.9574
NSF Rearing (s), IL-2	-0.171	9	-0.49	0.6244	0.9574
NSF Rearing (s), IL-3	0.029	9	0.083	0.9342	0.9784
NSF Rearing (s), IL-4	0.402	9	1.206	0.2278	0.9574
NSF Rearing (s), IL-5	-0.182	9	-0.521	0.6025	0.9574
NSF Rearing (s), IL-6	-0.185	9	-0.529	0.5966	0.9574
NSF Rearing (s), IL-9	-0.252	9	-0.729	0.4663	0.9574
NSF Rearing (s), IL-10	-0.01	9	-0.027	0.9784	0.9784
NSF Rearing (s), IL-12(p40)	-0.303	9	-0.884	0.3768	0.9574
NSF Rearing (s), IL-12(p-70)	-0.426	9	-1.286	0.1985	0.9574
NSF Rearing (s), IL-13	0.015	9	0.042	0.9669	0.9784
NSF Rearing (s), IL-17A	0.081	9	0.23	0.8181	0.9784
NSF Rearing (s), CXCL1	0.041	9	0.116	0.908	0.9784
NSF Rearing (s), CCL2	-0.328	9	-0.963	0.3355	0.9574
NSF Rearing (s), CCL3	-0.343	9	-1.012	0.3114	0.9574
NSF Rearing (s), CCL4	-0.028	9	-0.08	0.9365	0.9784
NSF Rearing (s), CCL5	-0.264	9	-0.764	0.4447	0.9574
NSF Rearing (s), TNFa	0.206	9	0.592	0.5542	0.9574

The correlation coefficients, degrees of freedom, *z*-values, and uncorrected and false discover rate (FDR) corrected *p*-values from the *z*-test correlation results of the 23 cytokines and chemokines assayed via Bio-Rad multiplex analyses with 11 behavioral outcomes not graphically displayed in Fig 6. Hypothesized correlation coefficient = 0. NISH, Novelty-induced sucrose hypophagia; NSF, Novelty-suppressed feeding.
